# Rootrainertrons: a novel root phenotyping method used to identify genotypic variation in lettuce rooting

**DOI:** 10.1186/s13007-025-01348-x

**Published:** 2025-03-02

**Authors:** Cara Wharton, Andrew Beacham, Miriam L. Gifford, James Monaghan

**Affiliations:** 1 Centre for Crop and Environmental Sciences, Harper Adams University, Newport, Shropshire TF10 8NB UK; 2 School of Life Sciences and The Zeeman Institute for Systems Biology & Infectious Disease Epidemiology Research, The University of Warwick, Coventry, West Midlands CV4 7AL UK

**Keywords:** Roots, Root phenotyping, Rooting assay, Lettuce, *L. sativa*

## Abstract

**Background:**

There is much interest in how roots can be manipulated to improve crop performance in a changing climate, yet root research is made difficult by the challenges of visualising the root system accurately, particularly when grown in natural environments such as soil. Scientists often resort to use of agar- or paper-based assays, which provide unnatural growing media, with the roots often exposed to light. Alternatives include rhizotrons or x-ray computed tomography, which require specialist and expensive pieces of equipment, not accessible to those in developing countries most affected by climate change. Another option is excavation of roots, however, this is time-consuming and near impossible to achieve without some degree of root damage. Therefore, new, affordable but reliable alternatives for root phenotyping are necessary.

**Results:**

This study reports a novel, low cost, Rootrainer-based system for root phenotyping. Rootrainers were tilted at an angle, in a rhizotron-like set-up. This encouraged root growth on the bottom plane of the Rootrainers, and since Rootrainers open (in a book-like fashion), root growth can be easily observed. This new technique was successfully used to uncover significant genotypic variance in rooting traits for a selection of lettuce (*L. sativa*) varieties across multiple timepoints.

**Conclusion:**

This novel Rootrainertron method has many advantages over existing methods of phenotyping seedling roots. Rootrainers are cheap, and readily available from garden centres, unlike rhizotrons which are expensive and only available from specialist suppliers. Rootrainers allow the roots to grow in substrate medium, providing a significant advantage over agar and paper assays.This approach offers an affordable and relevant root phenotyping option and makes root phenotyping more accessible and applicable for researchers.

**Supplementary Information:**

The online version contains supplementary material available at 10.1186/s13007-025-01348-x.

## Background

Climate change and a growing population increase demands on farming systems. Improving the understanding of root function in plants may help alleviate this pressure through improved crop management and generation of novel crop varieties [1–3]. The root system impacts on overall plant growth through nutrient and water uptake, anchorage, symbiotic interactions and abiotic stress tolerance, with better-adapted roots leading to improved plant health and yield [4]. There is evidence that domestication of crops has led to shallower root systems, thought to be due to breeders selecting plants under well irrigated and fertilised conditions, which may lead to reduced tolerance of transient droughts [5, 6]. Therefore, it is postulated that better understanding of root systems would enable improved crop varieties to be developed [7]. One example of focus is rapid rooting plants, which produce large functional root systems quickly, and have the potential to out-perform slower rooting plants by facilitating earlier access to nutrients and water in the soil [8]. Rapid rooting may also provide options for more sustainable agriculture, requiring reduced inputs of irrigation and fertilisers, as well as the potential to increase carbon storage in the soil through a greater root biomass [9]. Other targets of root improvements include: nutrient uptake [10]; reducing the metabolic cost of root growth (for example by increasing root cortical aerenchyma) [11]; disease resistance [12]; enhancing symbiotic interactions [13] and nitrogen-fixing nodule formation [14].

A challenge to be tackled in developing targeted root improvements is that root systems are intrinsically difficult to observe and study in their natural environment, as they remain hidden under the soil surface. All available methods for studying root systems have limitations. The current methods for studying root architecture can predominantly be classified into two groups. Those which are expensive, time-consuming or require specialist equipment, and those which are cheaper with high throughput potential, but which study roots on unnatural growing media. The first group includes techniques such as rhizotrons, minirhizotrons, ground-penetrating radar and x-ray computed tomography, which all offer excellent visualisation of roots but at cost or complexity [15–18]. The second group include techniques such as agar-, paper- and sand-based rooting assays, as well as observing root growth in hydroponic or aeroponic systems [15–18]. These assays can be cheap and high-throughput, however, the translatability and relevance of results from these assays to soil-grown plants is debatable [19]. It is also possible to perform root excavation or soil coring, however, these damage the fine roots, or enable analysis of only a small subset of root matter [20].

This paper presents a novel root assay method, adapting Rootrainers (which are cheap and readily available from commercial garden centres) into a rhizotron-like system, enabling observation of seedling roots grown on substrate. Although other studies have used Rootrainers for plant growth, for example, regarding the efficacy of Rootrainers vs traditional pots [21]; the cost effectiveness of using Rootrainers by growers [22] or for studying nutrient and water uptake of plants grown in Rootrainers [23], this is the first study, as far as we are aware, that uses Rootrainers to study root architecture.

This study tests a tilted Rootrainer set-up, analogous to a Rhizotron, to analyse rooting traits for different varieties of lettuce (*Lactuca sativa*). Strong root systems and rapid rooting genotypes are of particular importance to lettuce breeders due to the horticultural practice of raising seedlings in glasshouses before transplantation out into the field, which results in root damage (root pruning).

## Methods

### Plant material

Varieties used in this study are shown in Table 1, with seeds obtained from the Vegetable Genetic Improvement Network (VeGIN) Diversity Fixed Foundation Set (DFFS) collection held at the UK Vegetable Genebank at the University of Warwick [24]. The collection was generated using a double haploid process to ensure that all varieties are homozygous with genetically-identical seed within each accession.

**Table 1:** Lettuce varieties used in study, showing the accession, accession number and horticultural type (based on morphology)

Accession	Accession Number	Type	Variety name
CGN04628	LJ16162	Romaine	Kakichisha White
CGN09381	LJ16174	Iceberg	Gloire du Dauphine; Batavia Gloire du Dauphine
CGN09331	LJ16379	Butterhead	Grosse Brune Tetue; Bruine Trotskop
CGN04797	LJ16182	Cutting	4797
CGN13295	LJ16145	Butterhead	Cagraner Sommer
CGN05182	LJ14011	Iceberg	Great Lakes
Warwick LJ09001	LJ16411	Iceberg	Saladin
CGN05837	LJ16177	Cutting	White Lettuce

### Rootrainer set-up

**Fig. 1:**
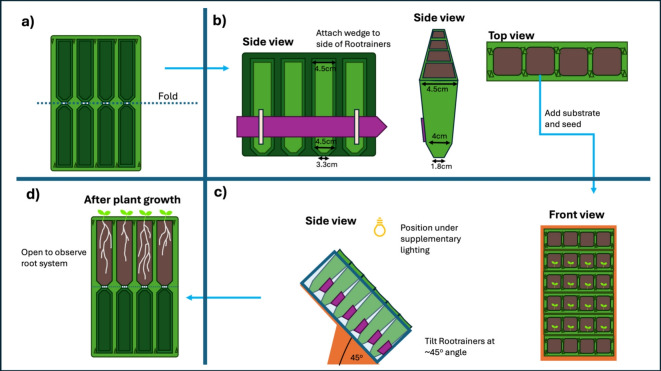
Depiction of how Rootrainers were set-up.** a** Unfolded Rootrainer.** b** The Rootrainers were folded in half, and a wedge (pink) stuck to each row (to compensate for the top of the Rootrainers being wider than the base and ensure a consistent angle of inclination). The cells were filled with substrate, the substrate wetted and the seeds sown.** c** The Rootrainers were arranged in boxes (each containing 6 rows of Rootrainers) and the boxes tilted to $45^{o}$, by resting on a wooden stand (orange). They were positioned under the supplementary lighting.** d** At each time point the Rootrainers were opened and the root systems observed

Maxi Rootrainers (20 cm deep, 350 cm$^{3}$ volume, Haxnicks, UK) were used for the experiment. These comprise of four cells, which are split in half vertically down the middle, joining at the base. The two halfs clip together to form a cell, in which substrate is placed (Fig. 1). The Rootrainers were set-up in a rhizotron-like fashion, being tilted at an angle of $45^{o}$, to encourage gravitropic root growth on the bottom surface of the Rootrainers (Fig. 1 and S1). Due to the tops of the Rootrainers being slightly wider than the base (dimensions given in Fig. 1), 2 mm wide wedges (large plant labels) were attached to the underside of each Rootrainer (Fig. 1 and S1), this ensured all the Rootrainers sat at the same angle. The Rootrainers were held in clear plastic boxes (42.5 x 33.0 x 17.0 cm, Tenlite Ventures Ltd, UK), leaning against a wooden support (custom made). Each plastic box contained six rows of Rootrainers (the top and bottom rows were left empty), and comprised of one block, with 2n of each variety (16 plants in total), randomly arranged within the block (each plant was assigned a random cell number using the sample() function in R without replacement). For visual explanation of the set-up view [25].

Two days prior to the first sowing, the Rootrainers were filled with substrate (‘Westlands Peat Free Multi-Purpose Compost with added John Innes’, Westlands, UK). The substrate was gently tamped down, and further substrate added to ensure the substrate was level with the top of the cells. The boxes were laid flat and water was added to the bottom of each box to 5 cm height, to wet the substrate, then removed after 20 min. The substrate did not noticeably slump during the experiment.

### Plant growth

The seeds were germinated on moist paper towels placed inside sealed plastic bags out of direct sunlight. The seeds were checked after 40 h, and seeds for which the radicle had emerged were randomly selected and sown into their randomly assigned Rootrainer cell (one per cell). The length of the radicle was measured, and the day of sowing was recorded as 0 DAG (days after germination). Rootrainers were then placed on a bench in a glasshouse bay at Harper Adams University (Shropshire, UK (52$^\circ$46’38.4”N 2$^\circ$25’41.1”W)) and grown from 24.07.2024 - 09.08.2024. Supplementary lighting was provided with white LED lights from 06:00-22:00 when ambient light levels dipped below 30 K lux. Vents were set to open when the temperature exceeded 17 $^{o}$C during the day and 10.5 $^{o}$C at night, or humidity exceeded 85 $\%$. A gapped light screen was programmed to close when 70 K lux ambient light was exceeded. The plants were watered every three days from below, by laying the box flat, adding water to the height of 3 cm to each box for 15 min, then transferring the Rootrainers into a fresh box (without water) and placing the box back at a 45$^{o}$ angle. For each block, a row of Rootrainers were weighed approximately 15 min after placing in the fresh box, with the mass recorded being used as a proxy to indicate that water levels were being restored to original levels (the growth of the young plants themselves were considered to have a very small, almost negligible, effect on the overall increase in mass). The temperature and humidity were recorded every 15 min by a TinyTag Plus2 TPG-4500 data logger (Gemini Data Loggers Ltd, UK) (Table 2).

**Table 2:** Conditions experienced during plant growth

Average daily minimum temperature	16.1 $^o$C
Average daily maximum temperature	33.6 $^o$C
Average temperature	24.4 $^o$C
Average daily minimum relative humidity	38.2 %
Average daily maximum relative humidity	84.3 %
Average relative humidity	59.7 %

### Phenotyping

**Fig. 2:**
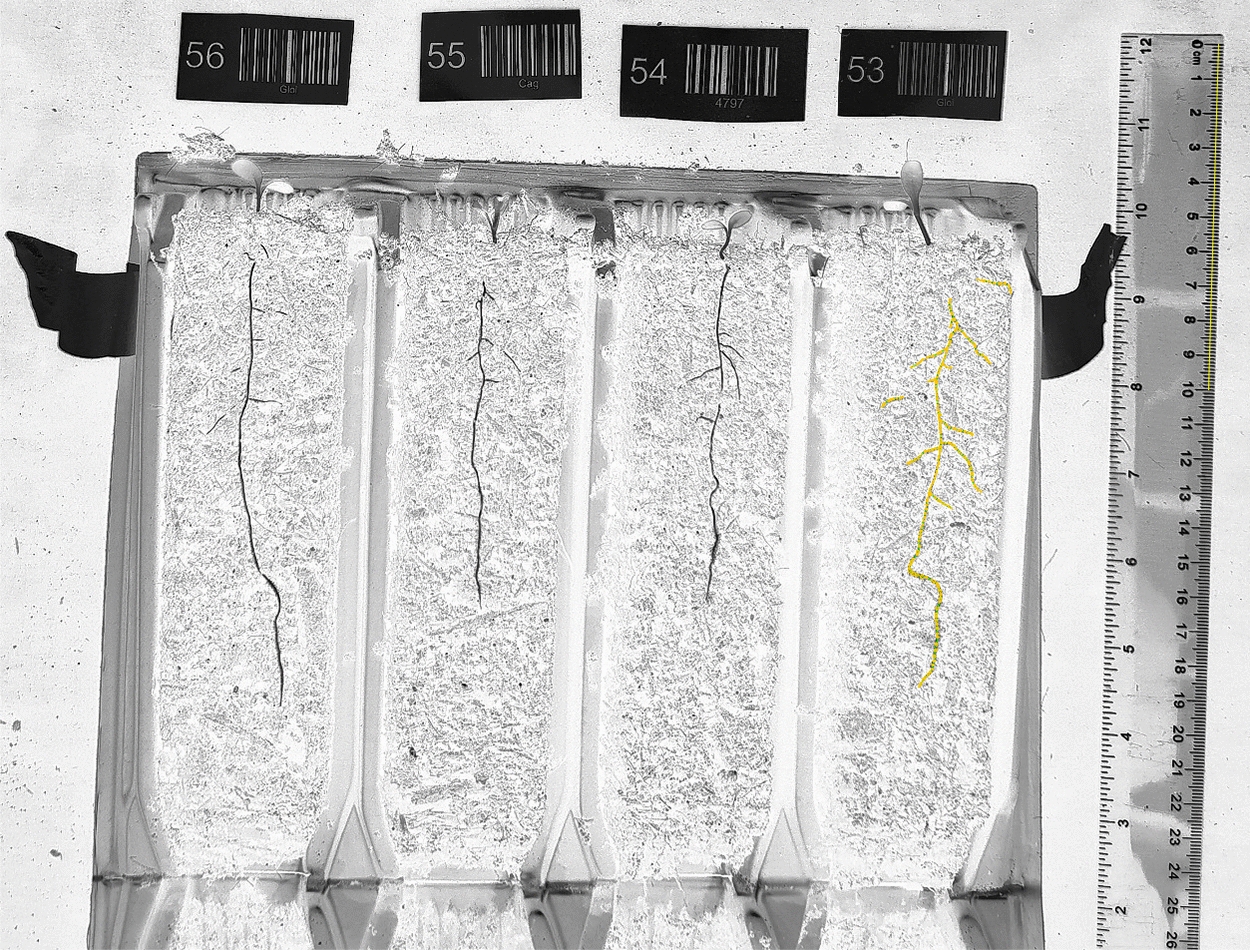
Determination of total root length. Root images were analysed using the SmartRoot plugin [27] within FIJI [26]. First the image was converted into an RGB (red, green and blue) stack, and the blue channel selected. The image was then inverted and opened in SmartRoot. The scale was set, and each individual root traced. The length of each root was extracted into a datafile, and all roots belonging to the same plant summed together to provide the total root length

**Fig. 3:**
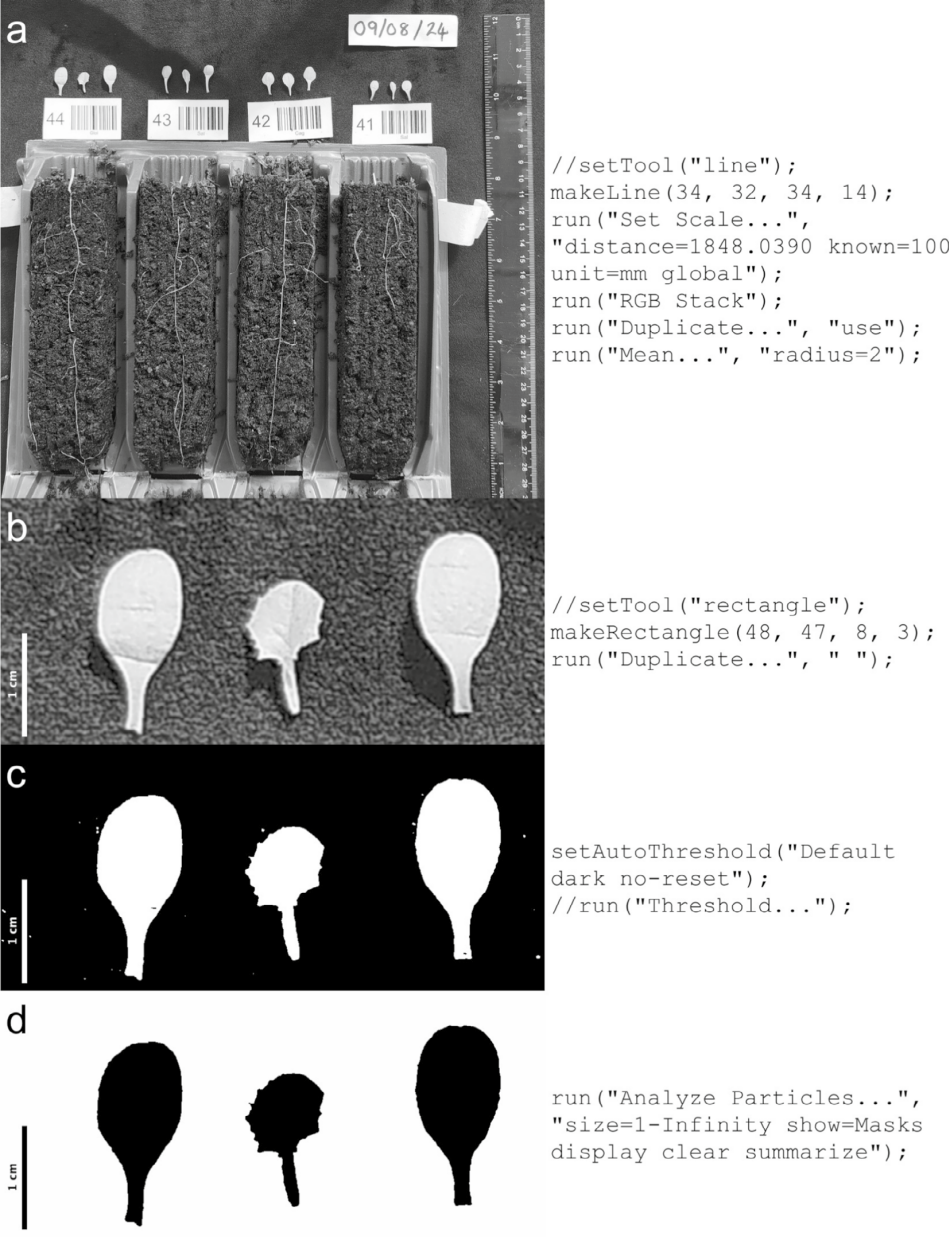
Determination of total leaf area.** a** The scale of the image was set against the ruler; the green channel of the image was used and then a filter applied to help smooth the background.** b** Leaves of one plant were cropped out for analysis.** c** The image was thresholded.** d** A mask was made of the thresholded image, removing any particles less than 1 mm in size; the masked image was then summarised, giving the total area. This analysis was performed in FIJI [26]. The corresponding code for each stage is shown to the right of the images

The Rootrainer cells were opened at 5, 7, 9, 12 and 16 DAG for root assessment. At the 5, 7 and 9 DAG timepoints the rooting depth (straight line from the soil surface to the depth of the deepest root) was measured using a ruler. After this timepoint, some of the plants had reached the bottom of the Rootrainers, and thus rooting depth is not reported. Additionally, at every timepoint, the roots of the seedlings were photographed from a fixed distance using a phone camera (108MP main camera, aperture = f/1.75, Redmi Note 13, Xiaomi, China), and the images were processed and analysed using the SmartRoot plugin in FIJI [26, 27], as shown in Fig. 2, to obtain the total root length. At 16 DAG the individual leaves were removed from the plant, placed on a flat surface, photographed from a fixed distance and analysed to obtain total leaf area using FIJI, as shown in Fig. 3.

### Analysis

In order to determine statistically significant differences in rooting depth and total root length between the varieties, mixed effect models were created and analysed [28, 29]. AIC (Akaike Information Criterion Statistics) values were used to determine which variables to include for each model, and histograms and DHARMa (Residual Diagnostics for Hierarchical Regression Models) plots of the model residuals were drawn to identify if the data adhered to the pre-requisite assumptions [30]. To improve adherence, the total root length data was square root transformed. To determine pairwise differences between the varieties, emmeans() was performed on the model, with Bonferroni p-value adjustments [31]. This was performed using the following code:
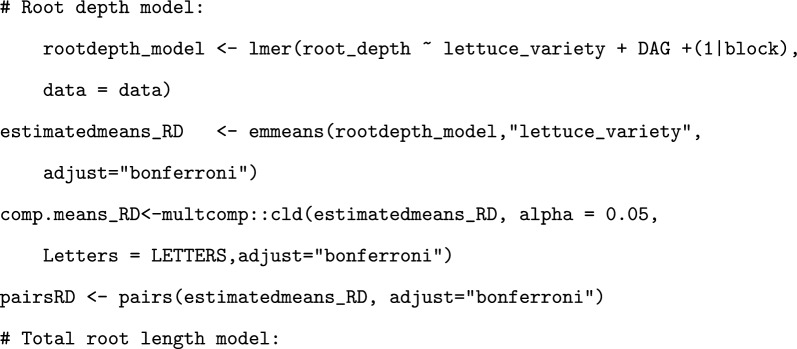

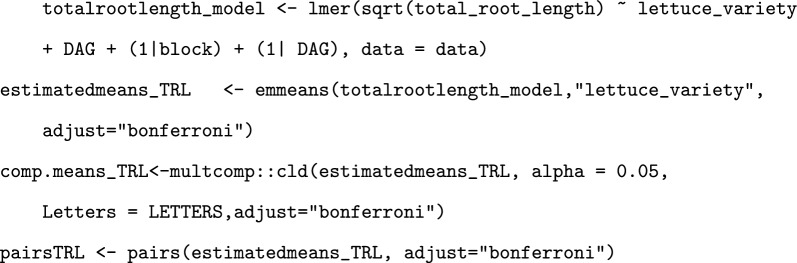


Root growth rate was calculated by dividing the growth change in total root length between the first and last timepoint by the number of days for each plant. This was analysed using a linear model, and pairwise comparisons made using emmeans() with Bonferroni adjustment. This was performed using the following code:
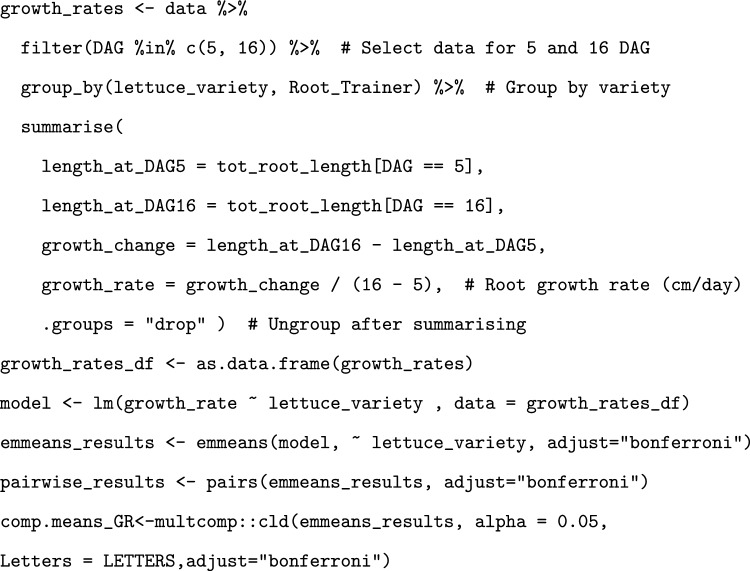


Additionally, a scatterplot was drawn to visualise correlations between the leaf area and total root length, and between initial radicle length and rooting depth [28, 32]. The regressions were analysed to get a Pearsons correlation coefficient using stat_cor() [33].

## Results

**Fig. 4:**
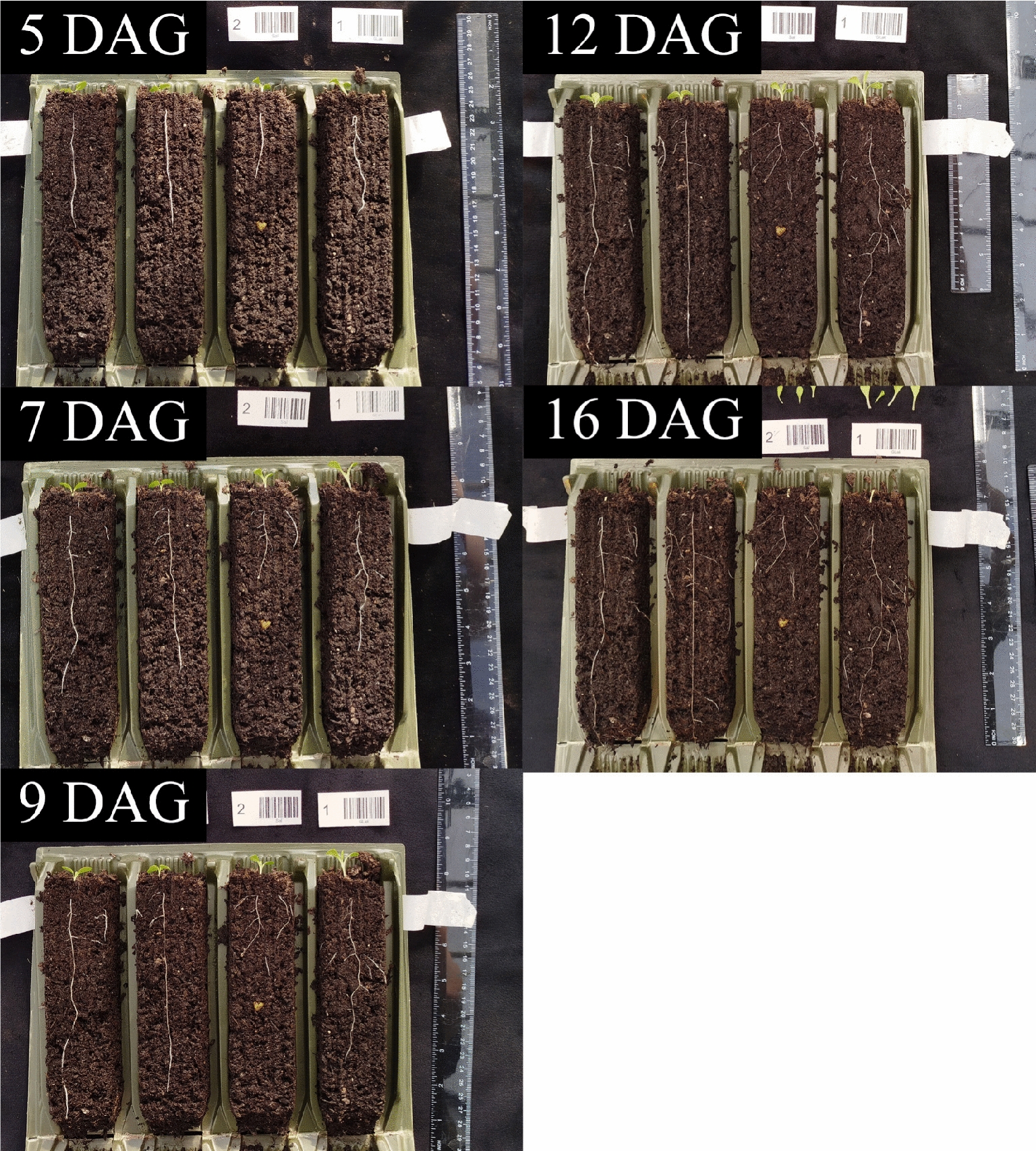
Growth of a subset of roots from the study across the different timepoints. DAG = Days After Germination

**Fig. 5:**
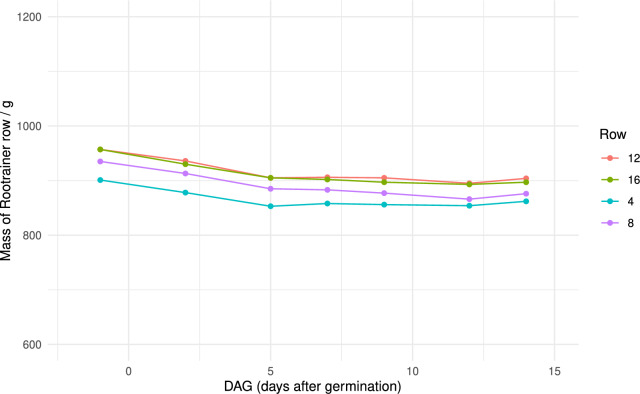
Mass of select rows of Rootrainers across the course of the experiment, as an indication of substrate water content

In order to compare lettuce total root system metrics between genotypes, 8 different varieties of lettuce (8n) were grown in tilted Rootrainers for 16 days. The system was simple to set up and the roots were easy to access and observe. Quality photos could be obtained of the root system at multiple timepoints, enabling valuable insights into the growth and development of the root systems (Fig. 4). The watering schedule was monitored by weighing one row of Rootrainers from each block to ascertain whether the mass, and hence the water content, reduced throughout the experiment. It was found that the although the water content initially decreased slightly, it then remained fairly stable, indicating that the watering regime was sufficient to return the moisture content to consistent levels (Fig. 5). It was also observed that the surface of the substrate did not dry out during the experiment.

### Initial radicle length had no impact on rooting traits at 5 DAG

**Fig. 6:**
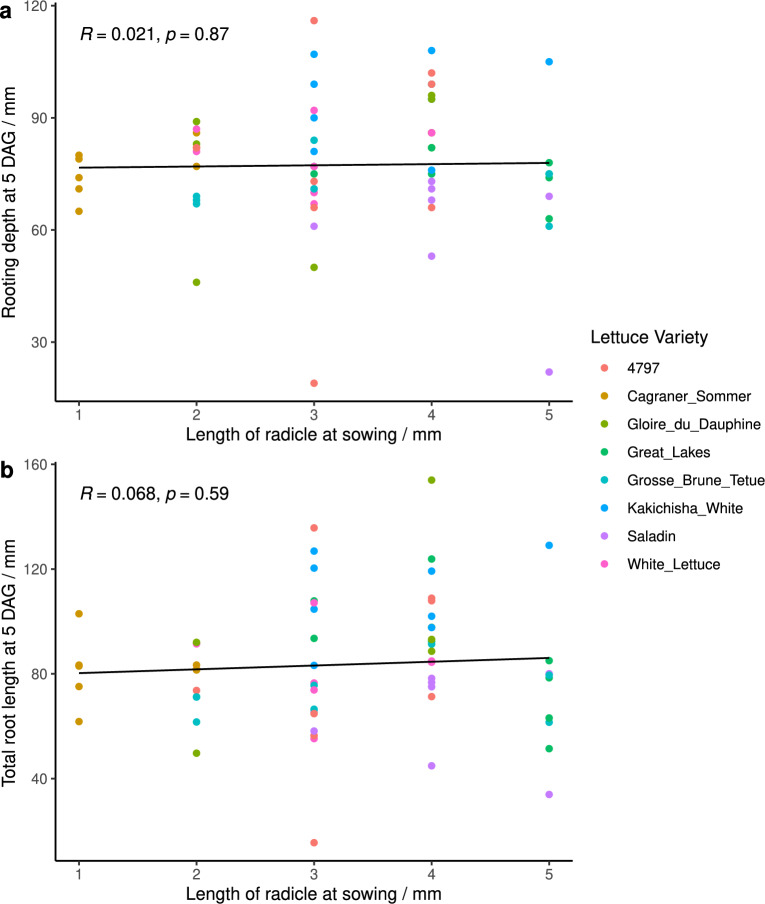
Initial length of radicle at sowing versus **a** rooting depth at 5 DAG and **b** total root length at 5 DAG. Regression lines are shown in black with Pearson’s coefficient (R) and p-value. Data points are coloured according to lettuce variety (see key). DAG= Days After Germination

There was no significant correlation between initial length of radicle at sowing, and either rooting depth (p-value = 0.87) or total root length (p-value = 0.59) at 5 DAG (Fig. 6a and 6b). The initial radicle lengths ranged from 1–5 mm, and were distributed across the different rooting depths seen at 5 DAG, which ranged from 19 to 116 mm. The only clear observation was that Cagraner Sommer had the shortest radicle lengths at sowing. However, this did not lead to shallower rooting depth at 5 DAG (Fig. 6a and 6b).

### Rooting depth showed genotypic variation

**Fig. 7:**
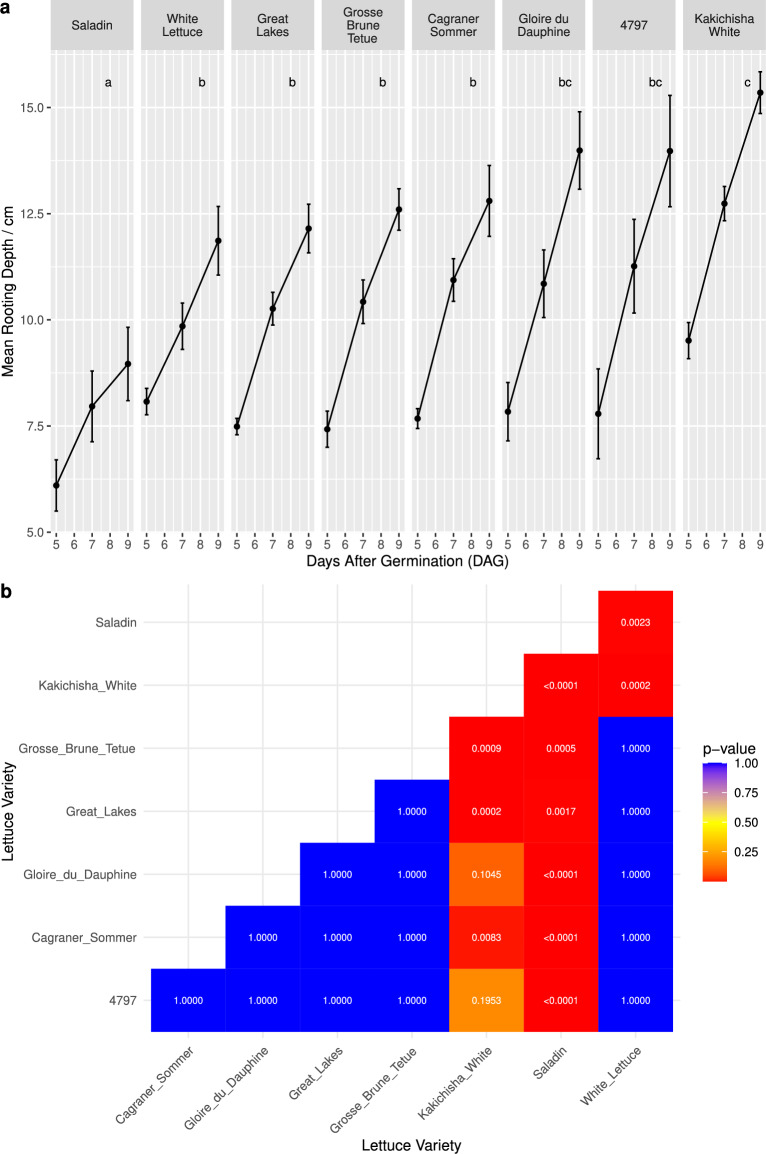
**a** Mean rooting depth for each variety at 5, 7 and 9 days after germination (DAG). Error bars indicate ± one standard deviation. Letters indicate significance groups.** b** Pairwise comparisons of the rooting depths between varieties, showing Bonferroni adjusted p-values

Analysis of the rooting depths of the different lettuce varieties across the timepoints revealed statistically significant differences between some of the varieties. Saladin was statistically shallower rooting than all other varieties, and Kakichisha White was significantly deeper than all varieties except Gloire du Dauphine and 4797 (Fig. 7). At 9 DAG, Saladin had reached a mean rooting depth of 8.96 cm, whereas Kakichisha White had reached 15.35 cm. Timepoints after 9 DAG were excluded, due to roots of the deeper growing varieties reaching the bottom of the Rootrainers, and thus not being able to grow down any deeper.

### Total root length showed genotypic variation

**Fig. 8:**
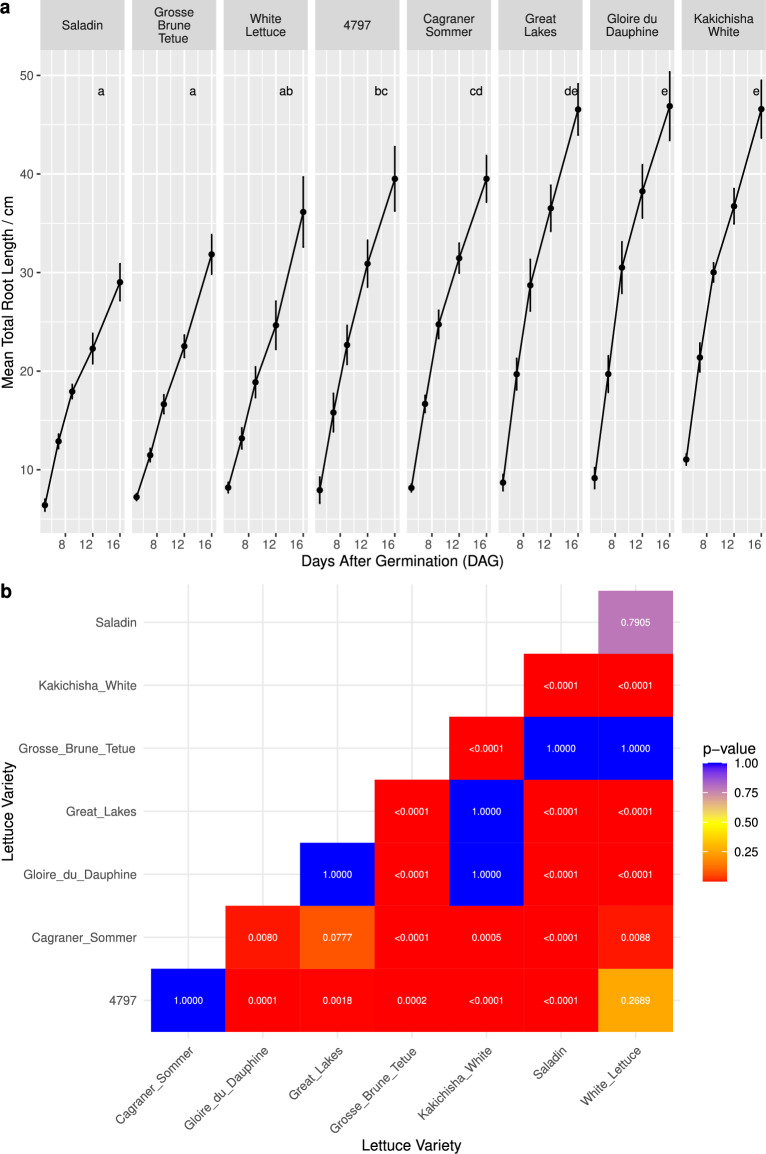
**a** Mean total root length at 5, 7, 9, 12 and 16 days after germination (DAG) for each lettuce variety. The error bars indicate ± one standard deviation. Letters indicate significance groups.** b** Pairwise comparisons of the total root length, showing Bonferroni adjusted p-values

Differences between varieties were also seen in total root length (Fig. 8a). For example, at 16 DAG, Saladin had a mean total root length of 29.02 cm, compared to Kakichisha White at 46.58 cm. Saladin and Grosse Brune Tetue had the smallest total root lengths, and were significantly smaller than all other varieties except for White Lettuce (Fig. 8b). The varieties with the largest total root lengths were Kakichisha White and Gloire du Dauphine, which were significantly larger than all the other varieties, except for Great Lakes (Fig. 8b).

### Root growth rate showed genotypic variation

**Fig. 9:**
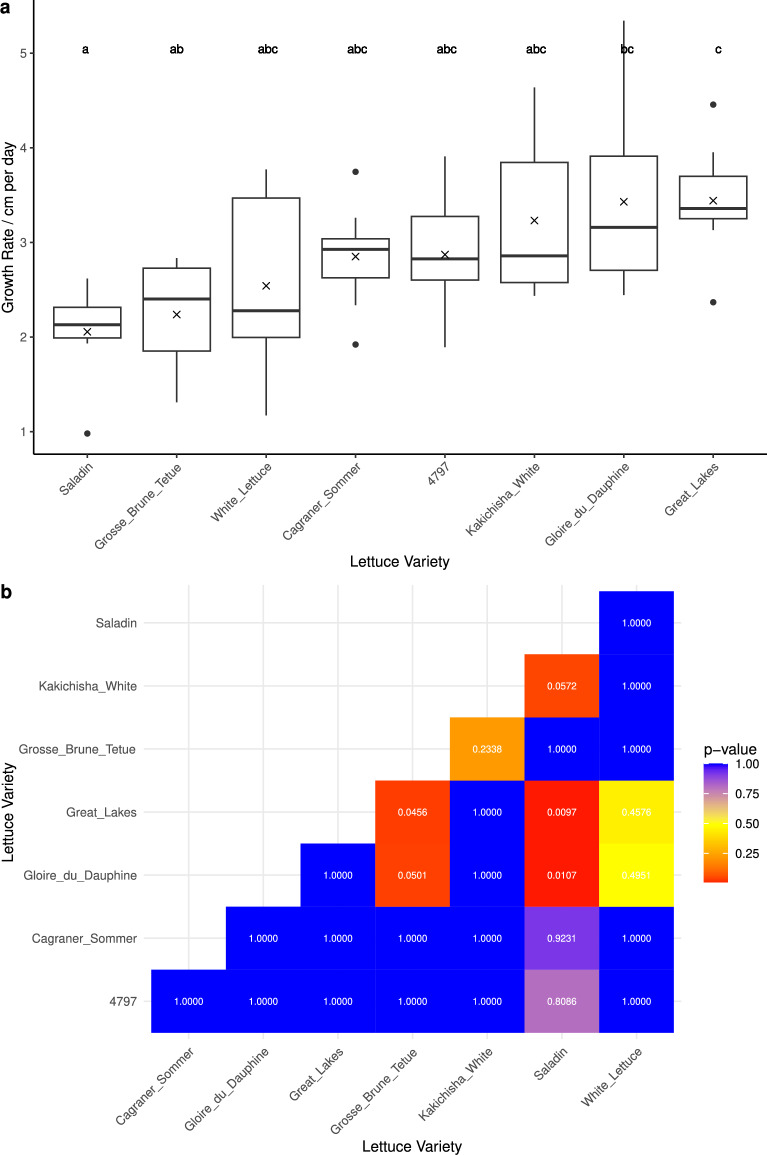
**a** Average growth rates of different varieties of lettuce over 11 days (DAG 5-16). The mean is represented by a cross, and the letters represent significance groups.** b** Pairwise comparisons of the root growth rate between varieties, showing the Bonferroni adjusted p-values

Saladin had the slowest mean root growth, at 2.06 cm/day, being significantly slower than Gloire du Dauphine (adjusted p-value = 0.0107) and Great Lakes (adjusted p-value = 0.0097) (Fig. 9). Great Lakes showed the fastest mean root growth rate, at 3.44 cm/day, being significantly faster than Saladin (adjusted p-value = 0.0097) and Grosse Brune Tetue (adjusted p-value = 0.0456) (Fig. 9). It should be noted that this represents a single average growth rate, and does not take into account that different root types may grow at different rates.

### Comparisons of rooting traits

**Fig. 10:**
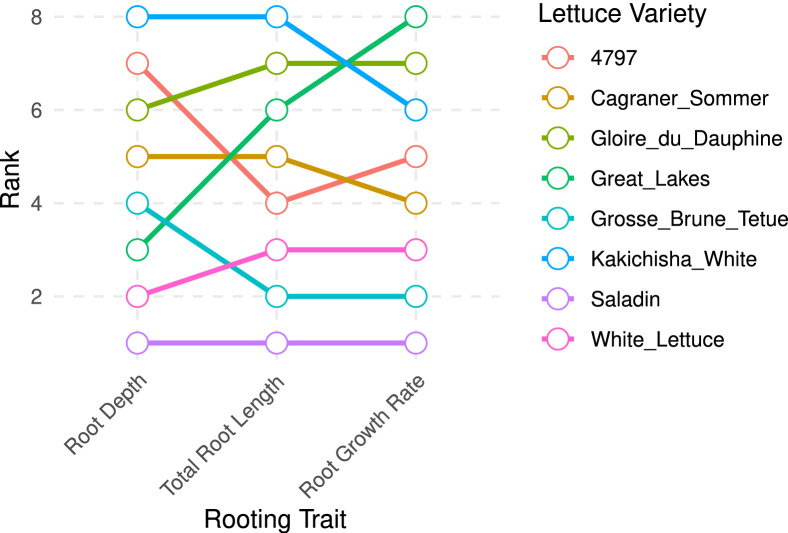
A comparison of the rooting traits displayed by each lettuce variety, visualised using ranks. A higher ranking indicates, respectively, a deeper/longer/faster-growing root system

The rooting traits of each variety were ranked using the mean values (Fig. 10). Saladin roots had the smallest or lowest values across all three traits (shallowest roots, smallest total root system and slowest growth rate). Whereas Gloire du Dauphine and Kakichisha White performed well for all three traits, yet they were out-performed by Great Lakes in terms of root growth rate. Great Lakes however, displayed a shallower rooting depth. Overall, there is some degree of agreement between the rooting traits, with 6 of the varieties having the same ranking for at least two of the rooting traits. Nonetheless, the ranks were not always consistent across the different traits. This is most apparent in Great Lakes which performed below average in terms of rooting depth, but was in the top three for root growth rate and total root length, suggesting a shallower, denser root system.

### Total root length and total leaf area showed a significant positive correlation

**Fig. 11:**
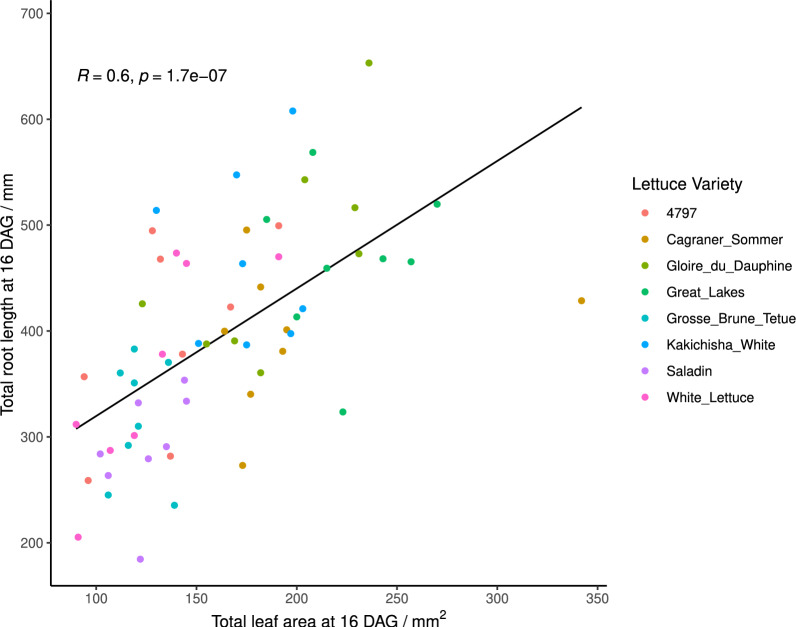
Total root length versus total leaf area at 16 days after germination (DAG). The regression line is shown in black with Pearson’s coefficient (R) and p-value. Data point colours indicates lettuce variety (see key)

When images of the leaves at 16 DAG were analysed to determine total leaf area and plotted against total root length at 16 DAG, it was found that they were significantly positively correlated with respect to each other (Fig. 11), with a Pearsons correlation coefficient of 0.60 (t=5.89, df=62, p-value = 1.71x 10$^{-7}$).

## Discussion

This study demonstrates that Rootrainers can be successfully used to assay root growth, allowing traits to be measured over multiple timepoints. Through comparing rooting phenotypes of different lettuce varieties, significant differences between lettuce varieties for both rooting depth and total root length were found, supporting previous work that genotype can play a significant role in lettuce root architecture [8, 34]. Additionally, the study revealed that initial radicle length at sowing had no significant correlation with rooting depth at 5 DAG, suggesting that the differences observed in rooting were not caused by the initial length of radicle. Furthermore, total root length and total leaf area displayed a positive correlation at 16 DAG. This opposes the traditional ‘optimal partitioning theory’ at the seedling stage in lettuce (which states that if more resources are allocated to the roots, then fewer are available for above ground growth, and vice-versa), which others have also raised doubts around [35]. The significance of these results validates that this Rootrainertron system can be used to successfully obtain useful and meaningful data for root architecture parameters grown over time.

The Rootrainertron system is cheap, making it accessible to a large number of scientists, which could enable increased research in this field. It is easy to set-up, has potential to be used with a wide range of species, and, due to the compact nature of the Rootrainers, has reduced space and resource requirements compared to e.g. traditional rhizotrons. This results in a system with potential for upscaling and use in high-throughput research. Additionally, it is a versatile system with options to study the effect of different environmental conditions, including the impact of different soil types, abiotic and biotic stresses. Moreover, the system allows direct access to the roots, enabling root samples to be taken from plants for transcriptomic and metabolic studies.

There are some limitations in the Rootrainertron system which also present in other methods, for example, both Rootrainers and rhizotrons require consideration when positioning lighting, due to the slanted nature of the systems. Similarly, that the shape may restrict root growth in the horizontal plane, is also found with rhizotrons and other 2D systems. However, there are some challenges faced solely by this method, including that there are constraints in the sizes of Rootrainers currently available (the largest Rootrainers found were Haxnicks Jumbo Rootrainers - width 7 cm x length 7 cm x depth 20 cm). Currently, this would limit Rootrainer assays to seedlings (for most species). The Rootrainers used in this study were ribbed, and all other available Rootrainers identified also had these ribs or grooves (vertical indentations along the Rootrainer cells). This was deemed not to cause an issue in the root imaging, however, may have resulted in alterations to the root architecture, with some companies claiming that they direct the roots downwards (when conventionally grown standing upright) [36]. Additionally, maintenance of soil moisture levels can be challenging, as the cells are not wide enough for moisture probes without risk of damage to the roots. In its supplied form, four cells are joined together in a row, making weighing each individual cell impossible. Therefore, watering from below was deemed most appropriate, and was acceptable for this type of study. However, the cells could be carefully cut apart and taped shut, if watering by mass was essential. Also, similar to all controlled environment rooting assays, the system could exhibit different substrate temperature and low soil compaction compared to field grown plants.

In terms of data collection, rooting depth and total leaf area were straightforward and easy to determine. However, not all the roots are visible, with some hidden under the substrate, despite tilting the Rootrainers to encourage root growth on the bottom surface. This will mean that, as with rhizotrons, total root length for the entire root system cannot be determined, instead visible roots were used as a proxy. It was assumed that the amount of hidden root for each plant will be more-or-less consistently proportional based on total root length. It has been shown in maize that roots observed in a rhizotron were representative of the total root system [37]. However, observed differences could be due in part to differing impacts or extent of gravitropic response between varieties, as genotypic variation in gravitropic responses have been observed in other species [38]. Additionally, roots which did not visibly branch off from the primary root could not be classified as lateral roots when tracing them. Therefore, not all the SmartRoot analysis functions could be used. This reflects the challenges faced when image analysing roots on substrate, also present in rhizotron assays. Indeed, in most ways this system could be considered as a simpler alternative to rhizotron assays, and unpublished data from previous rhizotron assays shows comparable results to those found here (S2). Rhizotrons are routinely used and accepted in root studies.

Although the use of this approach is best suited to less technical set-ups, further adjustments could be made to allow for it to be used with automated imaging tools. With these the positioning and alignment of the Rootrainers during imaging would need to be consistent throughout and more sophisticated camera systems considered. Here, SmartRoot has been used to manually trace the roots, however, there is an increasing number of image analysis tools available. The reader is encouraged to investigate these to determine which is best suited for their study and sample size. [15] provides an overview of root analysis softwares and [39] presents a review of softwares utilising artificial intelligence methods such as machine learning and deep learning. Another useful resource, providing a database of plant image analysis softwares, can be found at [40] [41].

Although there are adjustments which could be made to this approach which increase its scope, there are some situations for which this set-up would not be suited. For example, in high-cost robotic set-ups, the opening of the lids would most likely prohibit its use. Additionally, more in depth studies which require resolution of all roots, for example studying root branching, would not be suitable. It should also be noted that the act of opening the cells may affect rooting, and studies which are investigating highly sensitive traits, such as gene expression levels should plan their experiment accordingly.

Despite some limitations, this method has many benefits. The main advantages are that the roots are grown in natural growth media as opposed to e.g. agar or sand, and that it is cheap and easy to set-up, unlike 3D imaging approaches. This study shows that Rootrainertrons can be used to collect meaningful data which can be used to aid in plant breeding and developing crops with increased resilience to the changing climate. With rooting traits an important target trait in plant breeding, this novel rooting assay could be a useful tool for future developments, thereby facilitating root research and crop improvement.

## Conclusion

This paper verified a novel ‘Rootrainertron’ rooting assay, by successfully using it to identify genotypic variation in lettuce rooting. The study demonstrates that this Rootrainertron set-up could provide an accessible alternative for screening rooting traits of many species, with benefits over existing methods. It enables roots to be studied when grown in substrate, and therefore, has more relevance to studies in the field than agar- or paper- based rooting assays. The assay is cheaper than other substrate-based assays available, such as rhizotrons or x-ray computed tomography. Rootrainertrons enable the 2D root architecture to be observed and analysed, and the root system can be tracked over several timepoints and correlated to above ground growth. Additionally, the roots are available on the surface of the soil for tissue sampling, enabling, for example, correlation of gene expression with root phenotypes across many timepoints. Overall, this novel Rootrainertron method provides a simple but effective system for cheap analysis of roots grown in substrate, offering a great advance to root biologists and those interested in increasing horticultural and agricultural productivity.

## Supplementary Information

Supplementary file 1.

## Data Availability

The datasets used and/or analysed during the current study are available from the corresponding author on reasonable request.
