# Supplementary material for: Rootrainertrons: a novel root phenotyping method used to identify genotypic variation in lettuce rooting
Source: Plant Methods. 2025 Mar 2;21:29. doi: 10.1186/s13007-025-01348-x (PMC11872326; doi:10.1186/s13007-025-01348-x)
Supplement: Supplementary file 1 [file 13007_2025_1348_MOESM1_ESM.docx]

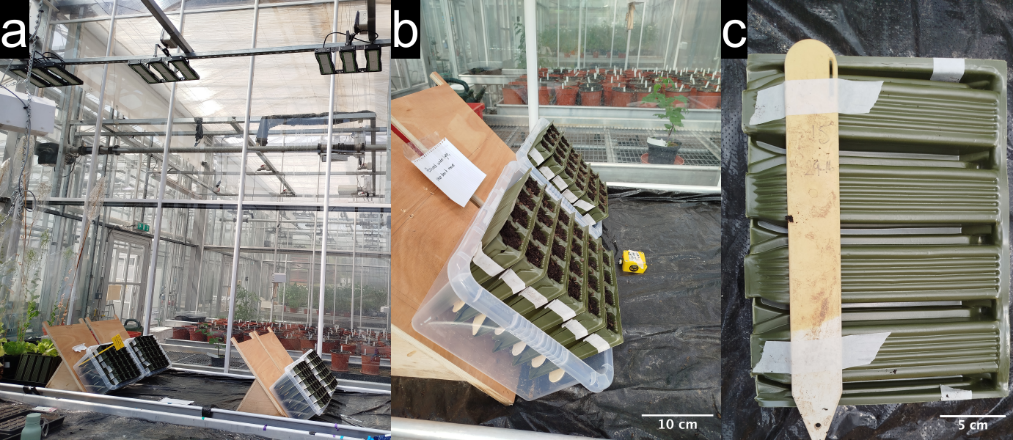


**S1:** Rootrainer experimental set-up. a) The positioning of the blocks beneath supplementary lighting in the glasshouse. b) Rootrainer placement within each box, supported on a custom stand at the required angle. c) A wedge was attached to each row of Rootrainers, to compensate for the top of the Rootrainers being wider than the base and ensure a consistent angle of inclination.


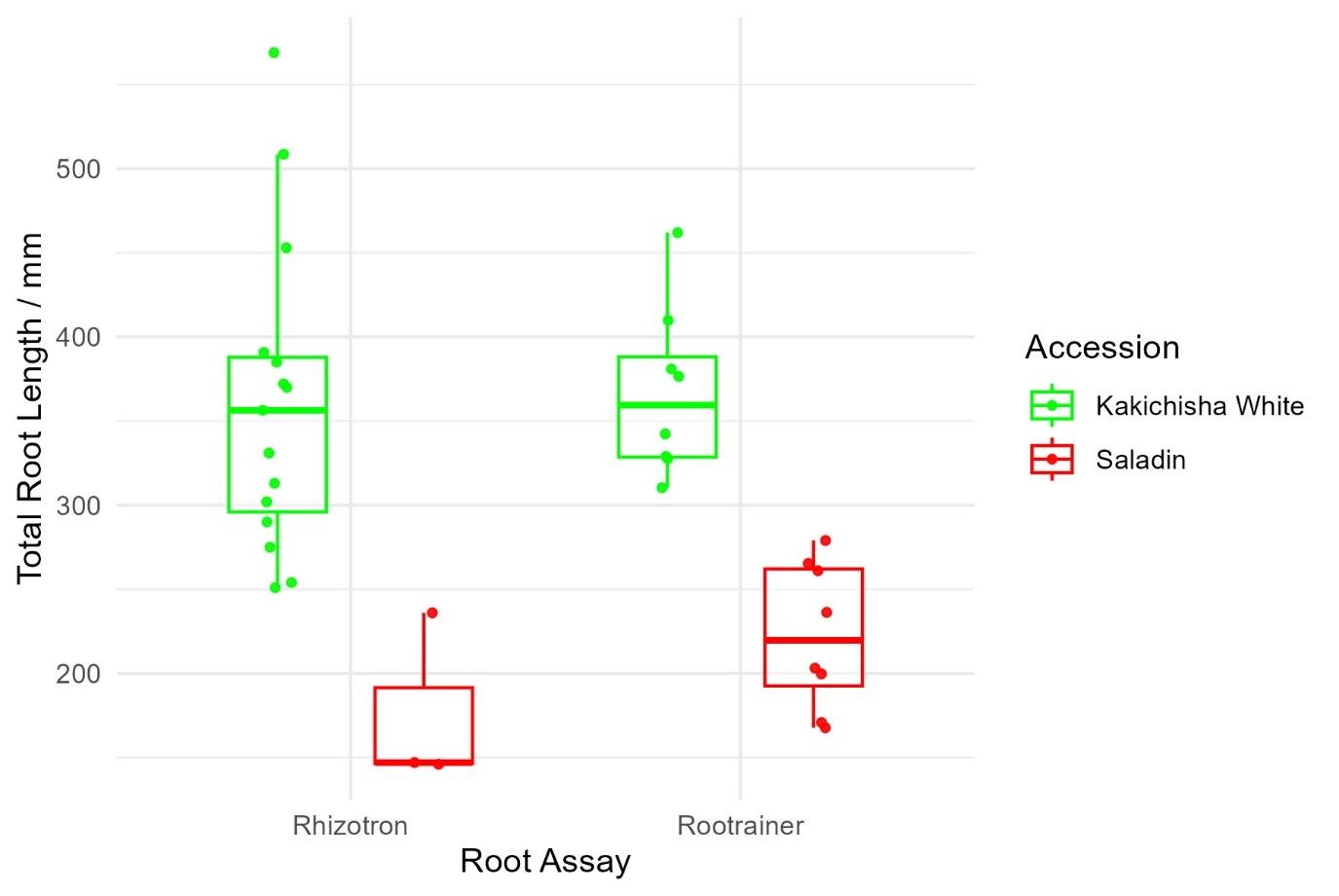


**S2:** Results of total root length of Saladin and Kakichisha White in Rootrainers compared to Rhizotrons. The assays were run at different times in glasshouse conditions, and thus were exposed to different weather patterns. The analysis was performed at a similar timepoint (12 DAG for Rootrainer assay, 12 DAG for first run of Rhizotron assay, 15 DAG for second run of Rhizotron assay).
